# Uncemented or cemented revision stems? Analysis of 2,296 first-time hip revision arthroplasties performed due to aseptic loosening, reported to the Swedish Hip Arthroplasty Register

**DOI:** 10.1080/17453674.2019.1624336

**Published:** 2019-06-03

**Authors:** Yosef Tyson, Ola Rolfson, Johan Kärrholm, Nils P Hailer, Maziar Mohaddes

**Affiliations:** a Section of Orthopedic Surgery, Department of Surgical Sciences, Uppsala University Hospital, Uppsala;; b The Swedish Hip Arthroplasty Register, Gothenburg;; c Department of Orthopedics, Institute of Clinical Sciences, Sahlgrenska Academy, Sahlgrenska University Hospital, Mölndal, Sweden

## Abstract

Background and purpose — Uncemented stems are increasingly used in revision hip arthroplasty, but only a few studies have analyzed the outcomes of uncemented and cemented revision stems in large cohorts of patients. We compared the results of uncemented and cemented revision stems.

Patients and methods — 1,668 uncemented and 1,328 cemented revision stems used in first-time revisions due to aseptic loosening between 1999 and 2016 were identified in the Swedish Hip Arthroplasty Register. Kaplan–Meier analysis was used to investigate unadjusted implant survival with re-revision for any reason as the primary outcome. Hazard ratios (HR) for the risk of re-revision were calculated using a Cox regression model adjusted for sex, age, head size, concomitant cup revision, surgical approach at primary and at index revision surgery, and indication for primary total hip arthroplasty.

Results — Unadjusted 10-year survival was 85% (95% CI 83–87) for uncemented and 88% (CI 86–90) for cemented revision stems. The adjusted HR for re-revision of uncemented revision stems during the first year after surgery was 1.3 (CI 1.0–1.6), from the second year the HR was 1.1 (CI 0.8–1.4). Uncemented stems were most often re-revised early due to infection and dislocation, whereas cemented stems were mostly re-revised later due to aseptic loosening.

Interpretation — Both uncemented and cemented revision stems had satisfactory long-term survival but they differed in their modes of failure. Our conclusions are limited by the fact that femoral bone defect size could not be investigated within the setting of the current study.

Uncemented revision stems are increasingly used in Sweden (Garellick et al. 2015), with revision being defined as removal or exchange of 1 or all parts of the prosthesis. In a register-based study, uncemented modular revision stems had a higher rate of re-revision compared with cemented revision stems (Weiss et al. 2011); however, mean follow-up was only 3.4 years for uncemented and 4.2 years for cemented revision stems. A retrospective study of 85 uncemented and 124 cemented revision stems (Hernigou et al. 2015) also observed increased risk of re-revision after use of uncemented fixation. In contrast, uncemented revision stems had a better survival in another retrospective study of 86 stems (Schmale et al. 2000), whereas Iorio et al. (2008) reported similar survival of uncemented and cemented revision stems, but the mortality was lower in patients operated with an uncemented stem. However, this study was also small, based on only 86 stems.

Thus, there is no consensus on whether uncemented or cemented revision stems are the best choice in femoral revision surgery. Bone defect size and varus remodeling, patient age and comorbidity, surgeon training and skills are all factors that are described to influence surgeon choice of fixation method (Della Valle and Paprosky 2004, Hartman and Garvin 2011). The reasons for re-revision in either type of stem fixation are also poorly delineated, even though this has been investigated for acetabular revision, describing differences between fixation methods (Mohaddes et al. 2013, 2015, Laaksonen et al. 2017), suggesting there might be a difference for stems as well. Mortality is also seldom reported. Our primary aim was to investigate how the risk of re-revision differs between uncemented and cemented revision stems. Secondary aims were to study how modes of failure differ between uncemented and cemented revision stems, and how the mode of stem fixation influenced short-term mortality after hip revision arthroplasty.

## Patients and methods

We designed a comparative cohort study on patients reported to the Swedish Hip Arthroplasty Register (SHAR), which is the oldest national THA registry in the world and has collected data on revisions from 1979 (Herberts et al. 1989). The reporting is voluntary but the completeness for primary THA is estimated to be 98%, and for revision THA 94% (Soderman et al. 2000).

**Figure 1.**
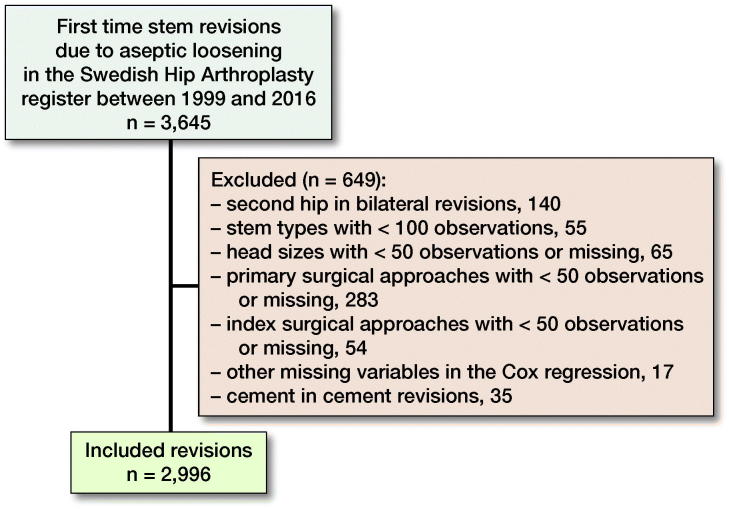
Flow chart of excluded stems.

First-time stem revisions due to aseptic loosening between 1999 and 2016 were identified. The rationale for choosing 1999 as the 1st year was the introduction of a more detailed recording of the implants in the registry. To reduce heterogeneity within the studied cohort the following exclusion criteria were applied: stem types with fewer than 100 observations; cement-in-cement revisions; head sizes with fewer than 50 observations; and surgical approaches at either index or primary surgery with less than 50 observations (Figure 1). Only cemented revision stems with a length of 165 mm or longer were included. Because only distally anchored uncemented revision stems are used, all stem lengths were included (Table 1, see Supplementary data). If both hips were revised, only the 1st revised hip was included in the study. Patients were divided into 2 treatment groups, those who received uncemented (n = 1,668) and those who received cemented revision stems (n = 1,328) during the index procedure. Primary outcome was re-revision for any reason, re-revision being defined as any subsequent revision, including but not limited to isolated stem revisions. Secondary outcomes were re-revision due to aseptic loosening, deep infection, dislocation, fracture, and other, and 90-day mortality. Follow-up started at index surgery (1st revision), and ended at re-revision, death, emigration, or December 31, 2016, whichever came first.  

### Statistics

Unadjusted survival was estimated using Kaplan–Meier analysis. Cox multivariable regression models were fitted to calculate hazard ratios (HR) with 95% confidence intervals (CI) with adjustment for age at index revision surgery, sex, indication for primary total arthroplasty, head size, concomitant cup revision (defined as acetabular shell revision or cup in cup revisions, whereas isolated liner exchanges were not included), and surgical approach at both revision and primary surgery. Because unadjusted survival curves deviated considerably from the assumption of proportionality, the regression model was divided into 2 time periods, choosing the dividing line at the time point where the unadjusted survival curves become roughly parallel. Schoenfeld residuals were calculated in order to assess whether model assumptions were met. The 1st year after index surgery was thus chosen as the 1st period, and the 2nd period described the 2nd to 13th years after index surgery. Sensitivity analyses were performed by calculating the HR only for patients operated with cemented stems at primary THA surgery (n = 2,815) in order to investigate whether fixation at primary surgery was associated with outcome, and by stratifying the study population into the following time periods for index revision surgery: 1999–2004 (n = 847), 2005–2010 (n = 1,176), and 2011–2016 (n = 973). The cohort was age stratified into 4 roughly equally large groups: age 67 and under (n = 750); age 68–73 (n = 703); age 74–79 (n = 793); and age > 79 (n = 750), followed by HR stratified for the different age groups. Descriptive statistics were used to study reasons for re-revision and early mortality. SPSS (IBM, version 22; IBM Corp, Armonk, NY, USA) and R Statistical Software (R Foundation for Statistical Computing, Vienna, Austria) were used for the calculations. 

### Ethics, funding, and potential conflicts of interest

Ethical approval was obtained from the Regional Ethics Review Board in Gothenburg, Sweden (decision 271-14). Financial support was received from the Health Care Committees in Region Uppsala and Region Västra Götaland. No competing interests were declared.

## Results

2,996 patients were included in the study, of whom 1,668 received uncemented revision stems and 1,328 cemented revision stems at the index procedure. Mean follow-up time among patients with uncemented revision stems was 5.5 years (SD 4.0), and it was 7.5 years (SD 4.5) for those with cemented revision stems. The sex distribution was similar in the 2 groups, but the head size differed, with uncemented stems more often receiving larger head sizes (Table 2).

**Table 2.** Demographic data of patients operated with uncemented or cemented revision stems. Values are frequency (%) unless ­otherwise specified

	Uncemented	Cemented
Factor	n = 1,668	n = 1,328
Women	713 (43)	580 (44)
Mean age at index surgery (SD)	72 (10)	74 (9)
Mean follow-up time, years (SD)	5.5 (4.0)	7.5 (4.5)
Mean time between primary and revision surgery, years (SD)	12.3 (6.0)	12.4 (5.7)
Diagnosis at primary THA		
Osteoarthritis	1,321 (79)	1,057 (80)
Fracture	90 (5.4)	86 (6.5)
Inflammatory disease	115 (6.9)	95 (7.2)
Condition after childhood disease	70 (4.2)	39 (2.9)
Other	72 (4.3)	51 (3.8)
Approach at index surgery		
Direct lateral	831 (50)	554 (42)
Posterior	837 (50)	774 (58)
Approach at primary THA		
Direct lateral	858 (51)	506 (38)
Posterior	810 (49)	822 (62)
Concomitant cup revision	1,258 (75)	1,328 (74)
Head size, mm		
22	90 (5.4)	112 (8.4)
28	841 (50)	870 (66)
32	570 (34)	271 (20)
36	167 (10)	75 (5.6)

Concomitant cup revision does not include only liner change.

**Figure 2.**
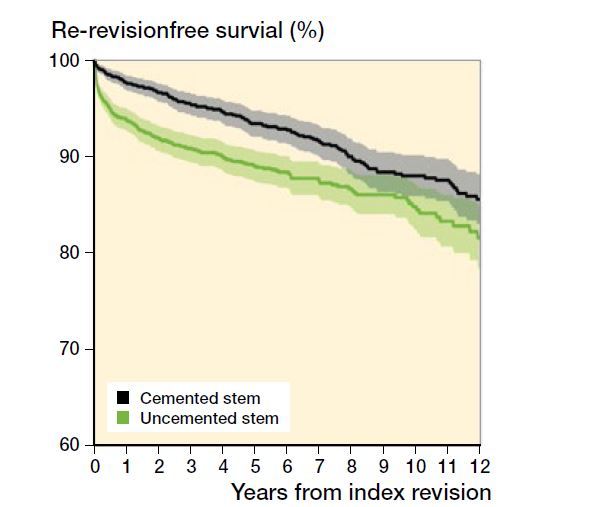
Unadjusted survival of uncemented and cemented revision stems.

Unadjusted 10-year survival was lower for uncemented compared with cemented revision stems (85%, 95% CI 83–87 versus 88%, CI 86–90) (Figure 2). The adjusted risk of re-revision for any reason was slightly higher for uncemented revision stems (HR 1.3, CI 1.0–1.6) during the 1st year after surgery (Table 3, see Supplementary data). From the 2nd year, the adjusted relative risk of re-revision for any reason for uncemented compared with cemented revision stems was 1.1 (CI 0.8–1.4) (Table 4, see Supplementary data). In the sensitivity analyses, the adjusted risk of re-revision for any reason of joints that had been primarily operated with cemented stems, and the risk of re-revision during different time periods were not substantially different from the risk that was estimated in our main analyses (Table 5, see Supplementary data). In patients 67 years and younger, the risk of re-revision was similar between uncemented and cemented revision stems (Table 6). Patients between 68 and 73 years also had a similar risk of early re-revision, whereas the risk of late re-revision tended to be lower for uncemented revision stems. In the group 74–79 years of age there was a slightly higher risk of re-revision for uncemented revision stems during both time periods. In patients older than 79 years, uncemented revision stems had a higher risk of early re-revision, but during the following period the risk was similar in the 2 groups. Taken together, this means that in patients up to 73 years of age uncemented stems perform as well as or better than cemented stems, whereas in patients 74 years or older, cemented stems perform as well as or better than uncemented stems.

**Table 6.** Risk of re-revision for uncemented revision stems, stratified by age groups at index revision surgery, with cemented stems as reference

	Year after index revision surgery	
	1st	Between 2nd and 13th
Patient age	HR (CI)	HR (CI)
< 68	1.0 (0.7–1.4)	1.0 (0.7–1.6)
68–73	1.0 (0.7–1.8)	0.6 (0.3–1.3)
74–79	1.4 (0.9–2.2)	1.5 (0.8–3.0)
> 79	2.4 (1.3–4.4)	0.9 (0.3–2.5)

HR (CI): adjusted hazard ratio and 95% confidence interval.

The distribution of reasons for re-revision differed between groups. Of the revision stems that were re-revised, more uncemented than cemented revision stems were re-revised due to dislocation and infection, but fewer of the uncemented revision stems were re-revised for aseptic loosening compared with cemented revision stems (Table 7).

**Table 7.** Reasons for re-revision for uncemented and cemented revision stems. Values are frequency (%)

Reasons for re-revision	Uncemented	Cemented
Aseptic loosening	49 (25)	65 (52)
Deep infection	38 (20)	18 (14)
Dislocation	65 (34)	17 (14)
Fracture	12 (6)	13 (10)
Other	29 (15)	13 (10)

The 90-day mortality was similar for patients operated with uncemented revision stems compared with those operated with cemented revision stems (12 [1%] versus 15 [1%]).

Further, we observed that patients who received concomitant cup revision during the index procedure had a lower adjusted risk of re-revision, both during the 1st year after surgery and between the 2nd and 13th year (HR 0.5, CI 0.4–0.7, and HR 0.5, CI 0.3–0.6) (Tables 3 and 4, see Supplementary data).

## Discussion

In this large cohort study analyzing 2,996 revisions, patients with uncemented revision stems had a higher overall risk of re-revision, especially among older patients. Uncemented stems were more often re-revised due to infection and dislocation, whereas cemented stems were more often re-revised due to aseptic loosening. The mortality was similar in both fixation groups.

Uncemented revision stems are increasingly used, but few studies have compared the survival of uncemented and cemented fixation. There is a controversy in the literature, describing uncemented fixation as inferior (Weiss et al. 2011, Hernigou et al. 2015), similar (Iorio et al. 2008), or better (Schmale et al. 2000) than cemented fixation. Revision THA is associated with worse patient-reported outcomes than primary THA and inferior implant survival (Patil et al. 2008, Singh and Lewallen 2009, Ong et al. 2010, Singh and Lewallen 2013), and the revision burden is estimated to double by 2030 (Kurtz et al. 2007).

In this study, the implant survival up to 13 years of uncemented and cemented revision stems was similar, with a higher re-revision rate for uncemented revision stems during the early postoperative period. This is in line with Weiss et al. (2011) who also describe higher early re-revision rates for uncemented revision stems. In our study, patients 68–73 years of age appeared to benefit from the decreased risk of long-term re-revision of uncemented revision stems. One could speculate that younger patients would benefit from use of uncemented stem fixation in 1st-time revisions because of decreased risk of late aseptic loosening, but we could not document such an advantage. Older patients on the other hand, with a shorter life expectancy, might benefit from the decreased risk of early re-revision with cemented revision stems. The increased risk of re-revision for uncemented revision stems in older patients might be due to compromised bone stock among these patients, increasing the risk for distal migration in uncemented revision stems, but this needs further elucidation. The risk of periprosthetic fracture was however not increased in this subgroup.

Uncemented revision stems were more often re-revised due to dislocation or infection. Similar findings have been reported from several national registries when analyzing the method of fixation in acetabular revisions (Mohaddes et al. 2013, 2015, Laaksonen et al. 2017). However, we recorded only the rate of re-revision, excluding dislocations treated without any surgical intervention, and also those treated with open reduction without removal or exchange of any implant parts. Several studies have reported dislocation rates between 3% and 19% for both uncemented and cemented revision stems (Hultmark et al. 2000, Haydon et al. 2004, Weiss et al. 2011, Pelt et al. 2014, te Stroet et al. 2015). In our study, larger femoral head sizes were more often used in uncemented revisions. Previous studies describe that larger head sizes decrease the risk for dislocation (Kosashvili et al. 2011, Garbuz et al. 2012, Wetters et al. 2013). It could be speculated that the increased risk of dislocation for uncemented stems would have been even higher if smaller heads had been used. It might also be that that the threshold to re-revise the proximal part of a modular uncemented revision stem is lower than re-revising a cemented revision stem due to dislocation, as suggested by other authors (Weiss et al. 2011). Also, older patients with cemented stems might have lower demand than younger patients with uncemented stems, potentially increasing the threshold to revise such patients due to dislocation. Of all the 194 uncemented revision stems that were re-revised, 46 (24%) included repositioning or exchange of only the proximal part of the stem, with or without concomitant cup revision, which supports this explanation. It could also be that the uncemented revision stems migrate distally and rotate into retroversion more frequently than cemented stems, especially when bone quality is compromised (Paprosky et al. 1999, Lakstein et al. 2010, Hernigou et al. 2015). Such migration might make the joint less stable and facilitate dislocation. The lower rate of re-revision due to infection after the use of cemented revision stems could be related to use of antibiotic loaded cement, a phenomenon that is described for primary total hip arthroplasty (Parvizi et al. 2008, Voigt et al. 2016). The difference in infection rate may also be due to selection bias. Cemented revision stems are more often re-revised due to aseptic loosening. Aseptic loosening is a late complication and the cemented stems have a longer follow-up time, which could in part explain this observation. Further, we do not have sufficient information on whether bone impaction grafting was used, which could have an impact on aseptic loosening. One could speculate that the cementation in a femoral canal devoid of trabecular bone could facilitate aseptic loosening. Also, fewer cemented revision stems were re-revised due to dislocation or infection, leaving more stems at risk of aseptic loosening. The pattern of cause-specific reason for re-revision of uncemented and cemented revision stems found by us has also been observed when comparing primary uncemented and cemented stems (Hailer et al. 2010, Gromov et al. 2015). Even though a larger proportion of uncemented revision stems received larger head sizes, they were more often re-revised due to dislocation. This seems to be in contradiction to previous studies where, in accordance with abundant literature, increasing head size was associated with a lower risk of revision due to dislocation (Hailer et al. 2012). We think that the increased re-revision rate might be explained by a lower threshold to exchange and lengthen or reposition the proximal part of an uncemented modular stem in cases with repeated dislocation, an intervention that is not easily available with well-fixed cemented non-modular stems (Paprosky et al. 1999, Lakstein et al. 2010, Weiss et al. 2011, Hernigou et al. 2015).

The groups had similar 90-day mortality, suggesting that cemented fixation does not increase the risk of short-term mortality. This is not in line with the observations of Iorio et al. (2008), but in that study mortality was measured over the entire study period and the difference observed could be caused by selecting older patients for cemented fixation. According to our observations, reduced early mortality is not a sustainable argument to use uncemented fixation, even if prospective, and preferably randomized comparisons are needed to support our findings.

Concomitant cup revision appeared to be associated with decreased risk of re-revision. This finding is difficult to interpret because this issue was not specifically addressed in our study, but it confirms previous reports from the SHAR (Karrholm et al. 2018). However, one could speculate that concomitant cup revision results in improved joint mechanics and might thus reduce the risk for dislocation (Bohm and Bischel 2004, Wetters et al. 2013). It has also been suggested that the primary THA cup is more likely to migrate and loosen than a revised cup (Mulliken et al. 1996, Bohm and Bischel 2004). On the other hand, the opposite situation has also been observed, namely that revision of the femoral component only is associated with lower risk of dislocation than if both components had been revised (Kosashvili et al. 2014). It is notable that three-quarters of procedures in both groups included a concomitant cup revision. We can only speculate on the reasons for this, but aseptic cup loosening, acetabular osteolysis, or simply wear of polyethylene cups or liners are frequent reasons for cup revisions during THA revision surgery. It is possible that Swedish surgeons have embraced findings from the SHAR indicating that concomitant cup revision lowers the rate of re-revision (Karrholm et al. 2018). Based on our findings, concomitant cup revision is advocated since it is associated with a reduced risk of re-revision both in the short and in the long term.

### Strengths and limitations

Although limited, this is the largest cohort study analyzing implant survival following uncemented and cemented revision stem fixation, using data from a national register with excellent coverage and high completeness.

This study has several limitations. First, there is no information regarding the presence or magnitude of any bone defects in either fixation group or the rationale for choosing either type of fixation, which is of course a major limitation. Hospitals tend to use either uncemented or cemented fixation (Figure 3, see Supplementary data); whether these choices are due to case mix or not is unclear, but it is unlikely that hospitals treat only patients with either large or small bone defects, and we believe that tradition might have an influence. It is also likely that surgeons in other parts of the world have different indications and usage patterns, which might decrease the generalizability of the findings outside of Sweden. In addition, we investigated only aseptic loosening as reason for re-revision, and it is possible that the findings would be different if other reasons, for example infection and periprosthetic fracture, were included. We did not report on cement in cement revisions since this has already been analyzed by our team (Cnudde et al. 2017). Proximally fixed uncemented revision stems were not investigated due to the fact that they are only in limited use in Sweden. The second limitation is lack of a population-based control group as regards mortality and no data on perioperative mortality; however, the risk of death on the operating table is low (Sierra et al. 2009, Ginsel et al. 2014). Finally, as in all register-based studies, there might be residual confounding.  

In summary, the 8-year risk of re-revision for uncemented stem fixation at first-time revision due to aseptic loosening was similar to that of cemented revision stems, but the different fixation principles differ in their modes of failure. Overall, both stem types offer excellent results.

## Supplementary data

Tables 1, 3, 4, 5, and Figure 3 are available as supplementary data in the online version of this article, http://dx.doi.org/10.1080/17453674.2019.1624336

All authors contributed to the design of the study, interpretation of the analysis, writing and revision of the manuscript. YT and MM conducted the statistical analysis.

The authors would like to thank the Swedish orthopedic surgeons and administrators reporting to the Swedish Hip Arthroplasty Register.

*Acta* thanks Richard N de Steiger and Wierd P Zijlstrafor help with peer review of this study.

## Supplementary Material

Supplemental Material
