# Supplementary material for: Uncemented or cemented revision stems? Analysis of 2,296 first-time hip revision arthroplasties performed due to aseptic loosening, reported to the Swedish Hip Arthroplasty Register
Source: Acta Orthop. 2019 Jun 3;90(5):421–6. doi: 10.1080/17453674.2019.1624336 (PMC6746274; doi:10.1080/17453674.2019.1624336)
Supplement: Supplemental Material [file IORT_A_1624336_SM4533.pdf]

Supplementary data

Table 1. Type of revision stems included in the study

Type of stem	Number of stems included in the study
Uncemented stems	
Link MP, Waldermar Link GmbH & Co. KG, Hamburg, Germany	902
Restoration Modular System, Stryker, Kalamazoo, MI, USA	233
Revitan Revision Hip System, Zimmer Inc, Warsaw, IN, USA	284
Wagner SL Revision Hip, Zimmer Inc, Warsaw, IN, USA	235
Cemented stems	
Exeter Hip System, Stryker, Kalamazoo, MI, USA	418
Lubinus SPII, Waldermar Link GmbH & Co. KG, Hamburg, Germany	716
Spectron, Smith and Nephew, Memphis, TN, USA	194

Table 3. Adjusted risk for re-revision 1st year after index surgery

Factor	HR (CI)
Uncemented stem (cemented ref.)	1.3 (1.0–1.6)
Age at index surgery (1 year)	0.97 (0.96–0.99)
Concomitant cup revision	0.5 (0.4–0.7)
Diagnosis at primary THA (OA ref.)	
Condition after childhood disease	0.9 (0.5–1.6)
Fracture	0.9 (0.6–1.4)
Inflammatory disease	0.7 (0.4–1.2)
Other	1.1 (0.7–1.9)
Female sex	0.8 (0.6–1.0)
Head size (22 mm ref):	
28	1.1 (0.7–1.7)
32	1.0 (0.6–1.7)
36	1.0 (0.5–1.9)
Posterior approach at index surgery [a]	1.1 (0.9–1.5)
Posterior approach at primary THA [a]	1.0 (0.8–1.4)

HR (CI): adjusted hazard ratio and 95% confidence interval
OA: osteoarthritis.
[a] Direct lateral approach ref.

Table 4. Adjusted risk for re-revision between 2nd and 13th year after index surgery

Factor	HR (CI)
Uncemented stem (cemented ref.)	1.1 (0.8–1.4)
Age at index surgery (1 year)	0.96 (0.95–0.98)
Concomitant cup revision	0.5 (0.3–0.6)
Diagnosis at primary THA (OA ref.):	
Condition after childhood disease	0.7 (0.4–1.5)
Fracture	0.7 (0.3–1.3)
Inflammatory disease	0.7 (0.4–1.3)
Other	1.0 (0.5–1.9)
Female sex	0.7 (0.5–0.9)
Head size (22 mm ref.):	
28	1.3 (0.7–2.3)
32	1.4 (0.7–2.6)
36	1.2 (0.4–3.4)
Posterior approach at index surgery [a]	1.0 (0.7–1.5)
Posterior approach at primary THA [a]	1.4 (0.9–2.1)

HR (CI): adjusted hazard ratio and 95% confidence interval
OA: osteoarthritis.
[a] Direct lateral approach ref.

Table 5. Additional sensitivity analysis

Factor	Year after index revision surgery 1st HR (CI)	Between 2nd and 13th HR (CI)
Only primary cemented THA	1.2 (0.98–1.6)	1.1 (0.80–1.5)
Revision stems inserted		
1999–2004	1.4 (0.90–2.1)	1.2 (0.75–2.0)
2005–2010	1.3 (0.91–1.9)	0.90 (0.58–1.4)
2011–2016	1.4 (0.86–2.3)	1.5 (0.89–2.4)

HR (CI): adjusted hazard ratio and 95% confidence interval for re-revision for uncemented revision stems after first time revision compared with cemented revision stems

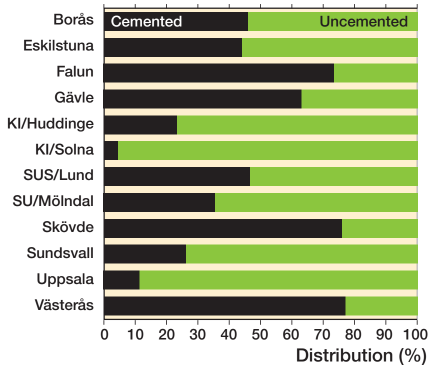

Figure 3. Distribution of usage of uncemented and cemented revision stems at hospitals that inserted more than 100 stems during the study period.
